# MHC class I diversity predicts non-random mating in Chinese alligators (*Alligator sinensis*)

**DOI:** 10.1038/s41437-018-0177-8

**Published:** 2019-01-22

**Authors:** Qun-Hua Han, Ru-Na Sun, Hai-Qiong Yang, Zhen-Wei Wang, Qiu-Hong Wan, Sheng-Guo Fang

**Affiliations:** 1 The Key Laboratory of Conservation Biology for Endangered Wildlife of the Ministry of Education and State Conservation Center for Gene Resources of Endangered Wildlife, College of Life Sciences, Zhejiang University, Hangzhou, 310058 China; 2 Changxing Chinese Alligator Nature Reserve, Changxing, 313100 China

**Keywords:** Molecular ecology, Ecological genetics

## Abstract

The major histocompatibility complex (MHC) has several important roles in kin recognition, pathogen resistance and mate selection. Research in fish, birds and mammals has suggested that individuals optimise MHC diversity, and therefore offspring fitness, when choosing mates. In reptiles, however, it is unclear whether female mate choice is based on genome-wide genetic characteristics such as microsatellite DNA loci, particular functional-trait loci (e.g., MHC) or both, and MHC's effects on mate choice remain relatively understudied. Herein, we used 13 microsatellite loci and two MHC class I loci to investigate female mate choice of Chinese alligators (*Alligator sinensis*) in the semi-natural condition. We also determined correlations between the MHC genotype of breeding males and male reproductive success. We found that MHC-heterozygous males harbour a greater reproductive success, which probably is the reason that these males are more preferred by the females than MHC-homozygous males. Furthermore, the MHC class I amino-acid distance and functional distance of true mating pairs were higher compared with those of randomly sampled pairs. Analysis of microsatellites revealed that, despite mate choice, females did not completely avoid inbreeding. These findings are the first evidence of MHC-associated mate choice in Chinese alligators, suggesting that females may adopt different mating strategies after assessing the MHC characteristics of potential mates.

## Introduction

Sexual selection has an important role in the evolutionary process by way of maintaining or enhancing population genetic variability (Milinski 2006; Piertney and Oliver 2006; Ruff et al. 2012; Kamiya et al. 2014; Gessner et al. 2017; Santos et al. 2017). Mate choice is a key component of sexual selection, typically manifesting as females selecting preferred males (Sommer 2005). Several studies have demonstrated that females selectively mate with males to increase offspring fitness (Candolin 2003; Penn and Potts 1999), and for direct (e.g., food, shelter and territory) or indirect benefits (high genetic quality) (Candolin 2003; Neff and Pitcher 2005).

In keeping with the importance of indirect benefits, multiple studies have indicated that the genetic characteristics of potential mates determine female mate choice (Strandh et al. 2012; Mariona et al. 2016; Rymesova et al. 2017). The major histocompatibility complex (MHC), which is a highly polymorphic, gene-rich family found in vertebrate genomes, is regarded as a good candidate for studying the genetic basis of mate choice (Klein 1986; Olsson et al. 2003; Sommer 2005; Sin et al. 2015), because MHC genes are crucial to adaptive immunity (Penn and Potts 1999), kin recognition (Manning et al. 1992; Ruff et al. 2012) and mate choice. MHC-associated mate choice has been studied for >40 years in various vertebrates, including mammals (Yamazaki et al. 1976; Huchard et al. 2013; Sin et al. 2015; Mariona et al. 2016), fish (Milinski et al. 2005; Agbali et al. 2010), reptiles (Olsson et al. 2003; Jaeger et al. 2016), birds (Juola and Dearborn 2012; Rymesova et al. 2017) and amphibians (Bos et al. 2009).

Three main hypotheses have been proposed to explain MHC-associated mate choice: good-gene hypothesis (Mays and Hill 2004; Cutrera et al. 2012), heterozygosity advantage hypothesis (Landry et al. 2001) and compatible-gene hypothesis (Zeh and Zeh 1996; Olsson et al. 2003; Puurtinen et al. 2009). According to the good-gene hypothesis, females prefer to mate with males that harbour a particular allele or genotype providing higher heritable qualities and increasing offspring fitness more than the average allele and genotype, in order to resist particular pathogens (Mays and Hill 2004; Penn and Potts 1999; Cutrera et al. 2012). In choosing a mate, females rely on phenotypic indicator traits as a proxy for a preferred genotype (Mays and Hill 2004). This hypothesis finds empirical support in the white-tailed deer (*Odocoileus virginianus*) (Ditchkoff et al. 2001) and Great Snipe (*Gallinago media*) (Ekblom et al. 2010).

The heterozygosity advantage hypothesis assumes that males with different MHC alleles are capable of fighting more pathogens than those with few MHC alleles (Landry et al. 2001; Mays and Hill 2004). Males that possess higher overall heterozygosity are generally attractive to females and appear to confer direct benefits through increased vigour (Mays and Hill 2004). In addition, mating with MHC-heterozygous males may produce MHC-heterozygous offspring (Landry et al. 2001). MHC-heterozygous males suffer from fewer parasitic infections, thus transmitting a lower parasite load to the offspring (Mariona et al. 2016). Correspondingly, female Talas tuco-tucos (*Ctenomys talarum*) (Cutrera et al. 2012), Gidgee skinks (*Egernia stokesii*) (Pearson et al. 2017) and grey wolf (*Canis lupus*) (Galaverni et al. 2015) tend to select MHC-heterozygous males.

The possibility that compatible-gene hypothesis may boost mate choice is attracting growing attention by researchers. Under this framework, females select males with MHC genotypes that are different from their own (Olsson et al. 2003; Neff and Pitcher 2005; Agbali et al. 2010). Importantly, it might provide a high diversity of MHC genotype (Apanius et al. 1997; Tregenza and Wedell 2000) and/or avoid detrimental effects from inbreeding for the offspring (Grob et al. 1998; Huchard et al. 2013). This hypothesis suggests that individual females can derive different advantages from mating with a given male (Puurtinen et al. 2009). There is sufficient evidence to illustrate mate choice involving gene compatibility, such as in great frigatebirds (*Fregata minor*) (Juola and Dearborn 2012), grey mouse lemurs (*Microcebus murinus*) (Schwensow et al. 2008), blue petrels (*Halobaena caerulea*) (Strandh et al. 2012), greater sac-winged bat (*Saccopteryx bilineata*) (Santos et al. 2016), Alpine marmots (*Marmota marmota*) (Ferrandiz-Rovira et al. 2016) and north American raccoons (*Procyon lotor*) (Santos et al. 2017).

The Chinese alligator (*Alligator sinensis*) is endemic to the freshwaters of China and is the most endangered of the 23 crocodilian species (Thorbjarnarson and Wang 1999). As a result of habitat loss and fragmentation, the wild population is declining at the rate of 4–6% every year, and fewer than 130 known individuals are present in the wild (Wang et al. 2011; Zhao et al. 2013). This endangered alligator population were not conserved well until the Changxing Chinese Alligator Nature Reserve (1979) and Anhui Research Center for Chinese Alligator Reproduction (1982) were found, and the number of captive individuals is ~15,000 at two reserve sites after decades of protection. Many research works have focused on the reproductive biology of the Chinese alligator (Thorbjarnarson et al. 2001; Chen 2003; Zhang et al. 2015; Zhao et al. 2013; Yang et al. 2017). Courtship and mating behaviour are an important part of reproduction (Chen 2003). According to our previous field observations for courtship and mating behaviour during the breeding season, female Chinese alligators show a tendency to mate with certain males (swimming or snout-touching together), while rejecting others (running away or diving into the water). However, no related research has focused on any crocodilian over the past 40 years (Kamiya et al. 2014), and the molecular mechanisms underlying mate choice of the Chinese alligator based on MHC variation remain unclear. Thus, MHC-based research on the Chinese alligator could expand the understanding of mating choice across vertebrates. In addition, as the Chinese alligator is an endangered species, this work could offer a better understanding of the evolutionary mechanisms influencing alligator populations and have great implications for their conservation and management, as well as for the management of other threatened species. Moreover, this study was conducted under semi-natural conditions, which could provide some insight into these cryptic, yet significant, biological phenomena.

In this study, we aimed to elucidate the molecular mechanisms of female mate choice by first investigating whether mating preferences are linked to MHC loci. The study employed 13 microsatellites and two MHC class I loci in the Chinese alligator. We tested three hypotheses based on the good-gene, heterozygosity advantage and compatible-gene hypotheses, respectively: first, females prefer males carrying a specific MHC I gene; second, females prefer heterozygous rather than homozygous males; and, finally, females prefer males that are genetically dissimilar from themselves regarding the MHC class I loci. We also examined whether the Chinese alligator is mates with relatives, and investigated potential correlations between the breeding-male MHC genotype and male reproductive success.

## Materials and methods

### Animal experiment ethics statement

All blood samples and umbilical cord tissues were collected in accordance with the guidelines approved by the Ethical Committee for Laboratory Animals of Zhejiang University, and all experimental protocols were also approved by the Committee.

### Sample collection and DNA extraction

We obtained blood samples of 182 adult Chinese alligators (123 females and 59 males) from three breeding ponds (Supplementary Table S1) at the Changxing Chinese Alligator Nature Reserve, China (30°93′N, 119°73′E). The three ponds are separated from each other by a dam and iron net. Hence alligators between the ponds cannot mate, but alligators in the same pond, having no physical barriers, can move freely to mate with each other during the breeding season. Umbilical cord tissues of newborn offspring from 105 clutches were also collected from 2011 to 2017. Tissue samples were stored at −20°C in 98% ethanol until DNA extraction. Genomic DNA extraction from all samples was conducted using the DNeasy Blood & Tissue Kit (Shanghai Generay Biotech Co., Ltd., China), following the manufacturer’s protocol.

### Microsatellite amplification and genotyping

Genotyping of subjects was performed at 13 neutral microsatellite loci (Supplementary Table S2, Table S3). The poPCR reaction mixture (10 μL) contained 1 μL of template DNA, 0.3 μL of forward and reverse primer, 3.4 μL of 2 × Taq PCR Master Mix and 5 μL of double-distilled water. The thermocycling protocol was 95°C for 5 min, followed by 32 cycles of 94°C for 30 s, 58°C for 30 s, 72°C for 30 s, and an extension of 72°C for 5 min. One of three fluorescent dyes (TAMRE, HEX or FAM) was used to label the 5′end of microsatellite forward primers. Negative controls included 1 μL of double-distilled water instead of the DNA template. Analysis of PCR products was conducted using an ABI 3130 Genetic Analyzer (Applied Biosystems Inc.). Allele size was determined using Gene Marker version 1.7.0, combined with visual analysis. To ensure accuracy in microsatellite genotyping, amplification and analysis were performed twice across all individuals and 1% genotyping error rate was allowed.

### MHC amplification and genotyping

Two MHC class I loci, I1327exon3 and I20exon2 (I1327e3: two alleles and I20e2: two alleles; Supplementary Table S2, Table S3), were amplified in a 10 μL reaction mixture containing 1 μL of template DNA, 0.3 μL of each primer, 3.4 μL of 2 × Taq PCR Master Mix and 5 μL of double-distilled water. The PCR reaction protocol was 95°C for 5 min, 32 cycles of 94°C for 30 s, 60°C for 30 s, 72°C for 30 s and 72°C for 5 min.

We used the PCR–single-strand conformation polymorphism (SSCP) technique to genotype each individual. In the SSCP process, each PCR product was added to 5 μL of 2 × loading buffer (0.025% xylene-cyanol, 0.025% bromophenol blue, 10 mm EDTA (ethylenediaminetetraacetic acid) and 98% formamide) and denatured at 95°C for 5 min, chilled on ice and then loaded onto a 12% non-denaturing polyacrylamide (Bio-Rad) gel (acrylamide: *N*, *N*′-methylenebis acrylamide = 37.5:1). Sequences were separated in 0.5 × TBE (tris-borate buffer) running buffer on the Dcode System (Bio-Rad) for 6.5 h at 10°C and 150 V. Finally, the gel was subjected to a 20-min treatment with 10% acetic acid, washed with dH_2_O and silver-stained to visualise the bands. The PCR–SSCP process was repeated at each locus to ensure a stable and consistent banding pattern.

### Parentage analysis

We conducted maximum-likelihood-based paternity analysis using Cervus 3.0.6 (Kalinowski et al. 2007). An error rate of 1% for mistyping, and for null allele or mutation was allowed. Paternity analysis was done using 95% confidence levels. In addition, the analysis was repeated in Colony 2.0 (Jones and Wang 2010) to reach 95% consistency between the two methods. Paternal genotypes for the known genotype of maternal–offspring clutches were reconstructed using Colony 2.0. MHC genotypes were used to examine the parentage results according to Mendel’s law of inheritance.

### Analysis of male reproductive success

The number of offspring in each clutch was considered the male reproductive success. We used the generalised linear mixed model with a Poisson distribution (logarithmic link) to assess the effect of male MHC genotype (MHC-heterozygote vs. MHC-homozygote) on reproductive success. The response variable was offspring number and the explanatory variable was defined as binary (“0” representing male MHC-homozygote and “1” MHC-heterozygote). The model was built using the glmer function of the LME4 package in R language using the following formula:

$$\begin{array}{l}{\mathrm{glmer}}\left( {{\mathrm{Offspring}}\_{\mathrm{number}}\sim {\mathrm{zygosity}}\_{\mathrm{status}} + \left( {1|{\mathrm{paternal}}\_{\mathrm{ID}}} \right),} \right.\\ \left. {\,{\mathrm{family}} = {\mathrm{poisson}}\left( {{\mathrm{link}} = {\mathrm{log}}} \right)} \right)\end{array}$$

### Statistical analysis

#### Mate choice hypotheses

##### Estimator used to test good-gene hypothesis

Each locus has two alleles. The male individual MHC allele frequency (AF) of each locus was used to assess whether females selected mates based on good gene.

##### Estimators used to test heterozygosity advantage hypothesis

Three estimators were used: (1) male individual microsatellite standardised heterozygosity (SH; Coltman et al. 1999), (2) *d*^2^ (genetic similarity between two alleles) and (3) male individual MHC heterozygosity (Ho). SH was calculated using the library Genhet of R language. Heterozygosity by locus (HL; Aparicio et al. 2006) and internal relatedness (IR; Amos et al. 2001) were also calculated as additional microsatellite heterozygosity indices, because these measures are highly correlated (all *R*^*2*^ *>* 0.95; Supplementary Table S4); only SH results are shown. Genetic similarity was calculated as *d*^2^ = 1/*n*∑^*n*^(*i*_a_ – *i*_b_)^2^, where *i*_a_ and *i*_b_ are the lengths of repeat motifs in the two alleles and *n* is the total microsatellite loci number. Ho was calculated as the proportion of heterozygous males among breeding males. SH and *d*^2^ were used to test whether female mate choice was based on genome-wide characteristics, while Ho was tested on the basis of MHC class I loci.

##### Estimators used to test compatible-gene hypothesis

Three pairwise estimators based on MHC class I were used: (1) allele sharing (Nsa) between pairs, (2) pairwise amino-acid distance (AAdist) and (3) pairwise amino-acid functional distance (AAfunc.dist). Values for allele sharing ranged from 0 to 2 (no allele, one allele or two alleles shared). The amino-acid distance of MHC alleles was calculated using MEGA 7.0, with the formula AAdist = DAB + DAb + DaB + Dab, where A, a, B, b represent the four alleles carried by pairs (Landry et al. 2001). For AAfunc.dist, the physiochemical properties of each amino acid were represented by five z-descriptors (Sandberg et al. 1998); a matrix between alleles was constructed and the Euclidian distance of all amino acids was calculated (Agbali et al. 2010; Huchard et al. 2013). The formula used to calculate AAfunc.dist is similar to the one used for AAdist.

#### Pairwise relatedness

Pairwise relatedness between mating partners was calculated using the Queller and Goodnight relatedness estimator (*R*_qg_; Queller and Goodnight 1989) in Co-ancestry (Wang 2011). Wang’s estimator (Wang 2002), as well as Lynch and Ritland estimator (Lynch and Ritland 1999), was also used. As the three neutral estimators of relatedness were highly correlated (all *R*^*2*^ *>* 0.79; Supplementary Table S5), only *R*_qg_ results are shown.

#### Randomisation tests

The random pairs were generated by sampling with replacement using Monte Carlo simulations. As the three ponds had different mating pairs, stratified sampling was performed. The details are as follows: a female and a male were randomly selected from the same breeding pool for pairing; the number of random-sampling pairs in each pond was consistent with the number of true mating pairs. The total number of random pairs from the three ponds was regarded as the total number of true mating pairs, and was used to calculate the mean value of the microsatellite and MHC genetic indices. We repeated the above method 9999 times to derive a distribution. Then we used a two-tailed test (significance set to *P* *<* 0.05) to compare the mean observed values of the true mating pairs with the sampling frequency distribution. Observed values were significant if they fell outside of the 95% confidence interval (CI). Statistical analyses and graphical representations were performed using Python (numpy, pandas, scipy and matplotlib modules).

## Results

### Parentage analysis

In all, 182 adults and 1684 offspring were genotyped successfully using both 13 microsatellite loci and 2 MHC class I loci. We reconstructed 27 paternal genotypes. Eighty-two parental pairs were assigned using 13 microsatellite loci. MHC genotypes were in keeping with Mendel’s law of inheritance, confirming the reliability of parentage analysis.

### No evidence for good-gene hypothesis

A comparison of observed MHC allele frequency (AF) per locus of breeding males and the the frequency distribution of randomly selected males revealed no significant difference (observed value_I1327e3_ AF = 1.268, *P* *=* 0.252; observed value_I20e2_ AF = 0.683, *P* *=* 0.837, Supplementary Figure S1).

### Preference mating with heterozygous males

**Fig. 1:**
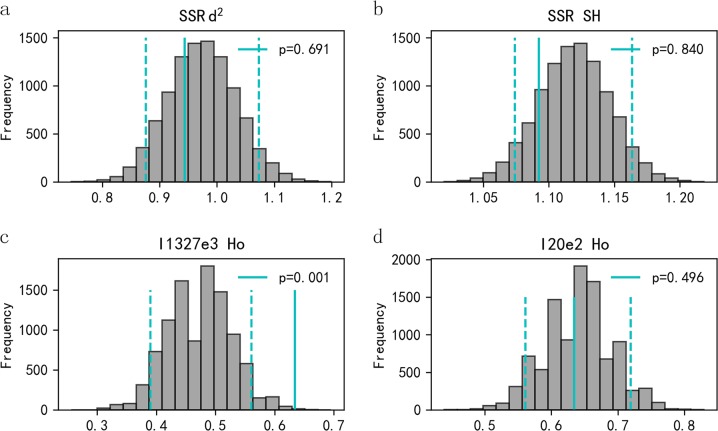
MHC-dependent mate choice tested with Monte Carlo simulations. Frequency distributions of mean *d*^2^, standardised heterozygosity (SH) and MHC heterozygosity (Ho), generated from 9999 Monte Carlo simulations of potential males (grey bars) compared with the observed values (solid vertical lines). Two-tailed 95% CI (dashed lines) indicate cutoffs for significant departures from randomly sampled males. **a** *d*^2^; **b** SH; **c** Ho of I1327e3 loci; **d** Ho of I20e2 loci

The *d*^2^ and SH (based on 13 microsatellites) of breeding males were compared with the distribution of randomly selected males. Breeding males and randomly sampled males did not differ significantly in *d*^2^ (observed value *d*^2^ = 9.437, *P* *=* 0.691, Fig. 1a) or SH (observed value SH = 1.092, *P* *=* 0.840, Fig. 1b). In contrast, breeding males had a higher Ho frequency for MHC I1327e3 locus than randomly sampled males (observed value_I1327e3_ Ho = 0.634, *P* *=* 0.001, Fig. 1c), but not for I20e2 locus (observed value_I20e2_ Ho = 0.634, *P* *=* 0.496, Fig. 1d).

### MHC-compatible gene mating

**Fig. 2:**
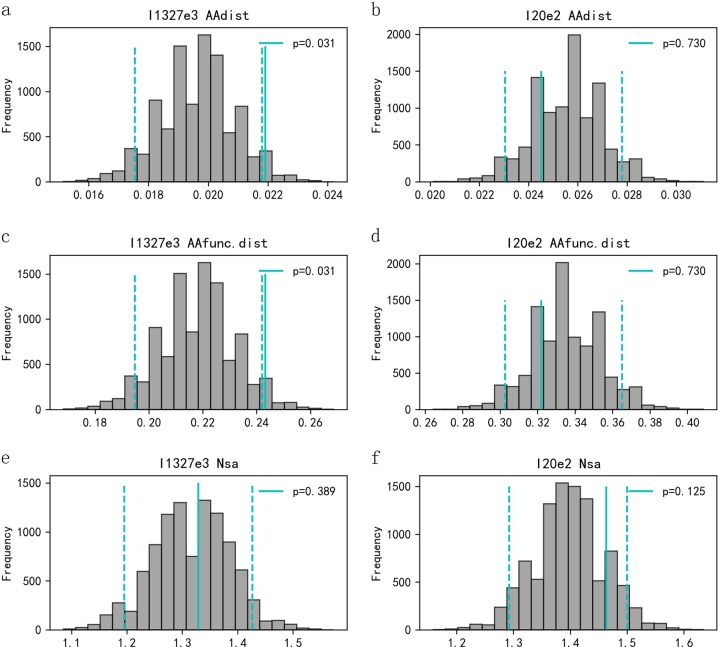
MHC-dependent mate choice tested with Monte Carlo simulations. Frequency distributions of mean MHC allele sharing (Nsa), pairwise amino-acid distance (AAdist) and pairwise amino-acid functional distance (AAfunc.dist), respectively, generated from 9999 Monte Carlo simulations of randomly sampled pairs (grey bars) compared with the observed value of true mating pairs (solid vertical lines). Two-tailed 95% CI values (dashed lines) indicate cutoffs for significant departures from random sampling pairs. **a** AAdist of I1327e3 loci; **b** AAdist of I20e2 loci; **c** AAfunc.dist of I1327e3 loci; **d** AAfunc.dist of I20e2 loci; **e** Nsa of I1327e3 loci; and **f** Nsa of I20e2 loci

True mating pairs had significantly higher AAdist for I1327e3 locus than randomly sampled pairs (observed value _I1327e3_ AAdist = 0.0218, *P* *=* 0.031, Fig. 2a). Pairwise AAfunc.dist for the I1327e3 locus also differed significantly (observed value_I1327e3_ AAfunc.dist = 0.242, *P* *=* 0.031, Fig. 2c). Neither AAdist nor AAfunc.dist differed significantly between true mating pairs and randomly sampled pairs for the I20e2 locus (observed value_I20e2_ AAfunc.dist = 0.0245, *P* *=* 0.730, Fig. 2b; observed value_I20e2_ AAfunc.dist = 0.322, *P* *=* 0.730, Fig. 2d). Nsa per locus between true mating pairs and randomly selected pairs did not differ significantly (observed value _I1327e3_ Nsa = 1.329, *P* *=* 0.389, Fig. 2e; observed value _I20e2_ Nsa = 1.463, *P* *=* 0.125, Fig. 2f).

### No obvious inbreeding avoidance

**Fig. 3:**
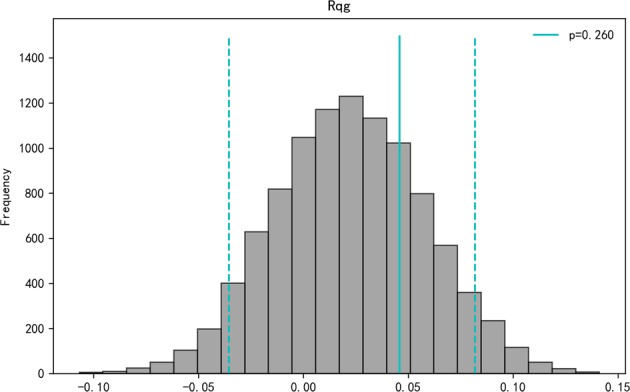
Effect of mate choice on pairwise relatedness. Frequency distributions of mean Queller and Goodnight relatedness (Rqg) generated from 9999 Monte Carlo simulations of randomly sampled pairs (grey bars) compared with the observed value of true mating pairs (solid vertical lines). Two-tailed 95% CI values (dashed lines) indicate cutoffs for significant departures from randomly sampled pairs

We compared Queller and Goodnight relatedness between true mating pairs and randomly sampled pairs and found no significant difference in pairwise relatedness (observed value *R*_qg_ = 0.046, *P* = 0.26, Fig. 3), indicating that females do not avoid mating with close relatives.

### Male MHC genotype and reproductive success

**Fig. 4:**
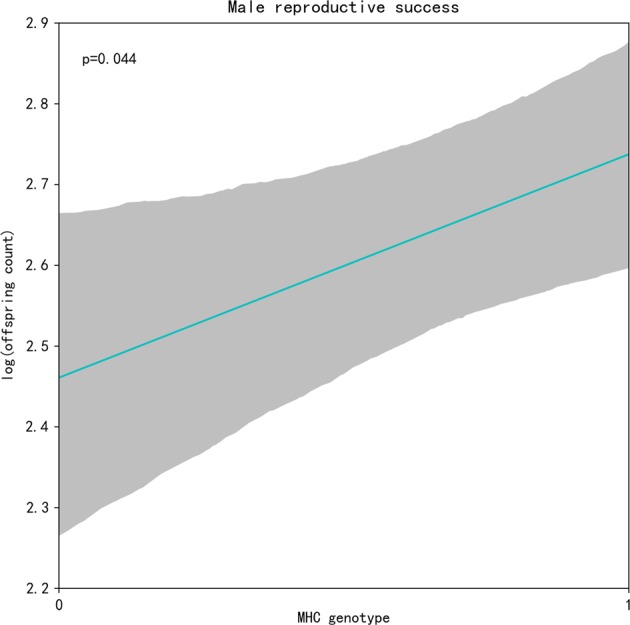
The relationship between offspring count and male MHC genotype tested with generalised linear mixed model (GLMM). Shaded areas represent two-tailed 95% CI, which were generated by 10,000 bootstraps

We investigated whether male MHC genotype exerts an effect on the number of offspring. Statistical significance was found for a correlation between male MHC genotype at I1327e3 locus and the number of offspring. In other words, heterozygous males had significantly higher reproductive success than homozygous males (*P* *=* 0.044, Fig. 4).

## Discussion

In our study, we found evidence that female Chinese alligators tended to mate with males exhibiting MHC heterozygosity and genetic compatibility. The lack of heterozygosity advantage among neutral markers (HS and *d*^2^; Fig. 1a, b) further suggested that MHC may be the target gene of female mate choice. The preference for MHC heterozygotes is in agreement with previous findings for wire-tailed manakin (*Pipra filicauda*) (Ryder et al. 2010), scarlet rosefinch (*Carpodacus erythrinus*) (Promerova et al. 2011), common lizard (*Zootoca vivipara*) (Laloi et al. 2011) and Talas tuco-tucos (Cutrera et al. 2012). Females may exhibit such a preference because heterozygosity is positively correlated with individual adaptability. For the Chinese alligators, the males provid no direct material benefits such as food, nest defence behaviours or parental care for the offspring (Chen 2003); hence the female may select heterozygous males in order to increase the genetic quality of offspring (Landry et al. 2001). In general, heterozygous males appear to be more capable of providing the indirect (fitness) benefits to females and their offspring (Mays and Hill 2004; Roberts et al. 2006; Mariona et al. 2016).

In support of the above-mentioned observation, we found that MHC-heterozygous males had higher reproductive success than MHC-homozygous males (Fig. 4). Galaverni et al. (2015) showed that the fitness values (average litter size) of grey wolf were positively correlated to the average heterozygosity of breeders at MHC loci. A similar result has also been reported for the Arctic Charr (*Salvelinus alpinus*) (Skarstein et al. 2005). One potential explanation for this higher performance of MHC-heterozygous males is a greater diversity of antigen-presenting molecules due to dissimilar MHC alleles, leading to broader pathogen resistance (Agbali et al. 2010). Consequently, females preferentially mate with genetically dissimilar males to increase the likelihood of producing heterozygous offspring (Skarstein et al. 2005).

The compatible-gene hypothesis was supported by the result that true mating pairs had higher pairwise amino-acid distance (Fig. 2a) and amino-acid function distance (Fig. 2c) than randomly sampled pairs at the I1327e3 locus. Female Chinese alligators choose males with compatible genetic characteristics that may be beneficial for several reasons. First, diseases due to external and internal parasites are prevalent in captive Chinese alligators (Zhao et al. 2015); this may be an alternative way to increase the MHC diversity of the offspring, which could improve their resistance to various pathogens or diseases (Apanius et al. 1997; Neff and Pitcher 2005). Second, our results showed that females did not avoid inbreeding (Fig. 3). The kind of female mate choice observed here is in accordance with other previous studies conducted both in the laboratory (sticklebacks, *Gasterosteus aculeatus*: Milinski et al. 2005; Chinese rose bitterling, *Rhodeus ocellatus*: Agbali et al. 2010; mandrill, *Mandrill ussphinx*: Setchell et al. 2010) and in the field (sand lizard: Olsson et al. 2003; Schwensow et al. 2008; grey mouse lemurs: Huchard et al. 2013; great sac-winged bats: Santos et al. 2016). For example, a study of wild grey mouse lemurs showed that the amino-acid distance of fathers to females is higher than that of randomly assigned males (Schwensow et al. 2008).

In contrast to our findings, some species show no evidence of MHC-dissimilar mating, including great tits (*Parus major*) (Sepil et al. 2015), great reed warbler (*Acrocephalus arundinaceus*) (Westerdahl 2004) and common yellowthroats (*Geothlypis trichas*) (Bollmer et al. 2012). Still others actually exhibit a preference for similar MHC, such as house sparrows (*Passer domesticus*) (Bichet et al. 2014), European badgers (*Meles meles*) (Sin et al. 2015) and fish (*Poecilia reticulate*) (Clelia et al. 2015). In addition, there are conflicting reports about MHC-based mate choice for species such as Atlantic salmon (*Salmo salar*) (Consuegra and de Leaniz 2008; Promerova et al. 2017).

Preferential mating for heterozygosity advantage and compatible genes is not mutually exclusive, as females can simultaneously assess both characteristics according to a hierarchical, nested rule (Mays and Hill 2004). Research on mice supports this mechanism: female mice first favoured certain males based on urinary scent marks, and then selected males with the greatest MHC dissimilarity among the initially preferred individuals (Roberts and Gosling 2003). For Chinese alligators, genome sequence has unravelled that they harbour a genetic basis of a robust sensory system (Wan et al. 2013). Therefore, female Chinese alligators may select males with high or medium heterozygosity, and then fine-tune the choice by considering genetic compatibility. Similar findings have also been reported in giant panda (*Ailuropoda melanoleuca*) (Zhu 2014) and golden snub-nosed monkey (*Rhinopithecus roxellana*) (Song 2016).

In the semi-natural condition the alligators congregate because of bellowing behaviour during the mating season, so the chances of the female searching for and selecting the potential mating male are high (Wang et al. 2006). Previous studies have shown that MHC has a role in kin recognition and inbreeding avoidance (Manning et al. 1992; Grob et al. 1998; Ruff et al. 2012). However, here we found that Chinese alligator females did not avoid close relatives when selecting males (Fig. 3). This finding is surprising given the widely acknowledged adaptive importance of inbreeding avoidance (Pusey 1996; Bull and Cooper 1999; Hu et al. 2017; Huchard et al. 2017). In our study, 182 adult alligators are from only 11 wild founders, suggesting that breeding with close relatives is unavoidable. Thus, being left with no other suitable potential mates, the females have no choice but to mate with relatives to maximise the fitness and survival rate of offspring, regardless of the risk of inbreeding depression. Multiple studies have reported that inbreeding avoidance is absent if the benefits outweigh the costs of inbreeding avoidance (Kokko and Ots 2006; Jamieson et al. 2009; Szulkin et al. 2013; Huchard et al. 2017). For example, the dispersal behaviour of captive populations is limited and encounters between close relatives are easy to occur; hence, avoidance of close breeding may result in delaying or missing opportunities for breeding (Keller and Arcese 1998). Studies also show that mating with a close partner could be advantageous, as it increases the parent’s inclusive fitness (Waser et al. 1986; Kokko and Ots 2006). However, for many threatened species, it is impossible to have a natural ‘built-in’ mechanism for avoiding inbreeding (Jamieson et al. 2009).

Our study has some notable limitations. First, although we found evidence that female Chinese alligators prefer males with heterozygous and genetically dissimilar MHC I loci, the direct phenotypic traits for recognition of partners remain unknown and are difficult to be observed. Second, the number and density of the wild population are, respectively, less and scattered, which may make it more or less likely that the pattern of mate choice is different from that of the semi-natural population in our study. Third, although our results show that MHC-heterozygous males have increased reproductive success (offspring number) compared with MHC-homozygous males, we cannotrule out the possibility of the effect of potential confounding factors such as nesting environment or raining on the reproductive success.

In conclusion, this is the first study to describe MHC-associated mate choice in any crocodilian in semi-natural condition. Our data support the heterozygosity advantage and compatible-gene hypotheses, which contributes to our knowledge of MHC-based mate choice in reptiles. However, our study gives rise to additional unsolved questions regarding MHC-mediated mate choice, which deserves future study in this endangered species. For instance, colour, olfaction, acoustics and other morphological traits are reliable signals of MHC genes (Penn 2002; Olsson et al. 2003; Overath et al. 2014; Garamszegi et al. 2018), suggesting fruitful avenues of further research to understand whether such traits also reflect MHC in Chinese alligators. Intriguingly, genome sequence analysis has demonstrated robust olfactory, visual and aural systems for Chinese alligators (Wan et al. 2013). These phenotypic cues may be the means through which Chinese alligators recognise the appropriate MHC identity of potential mates.

### Data archiving

Sequences have been submitted to GenBank under accession numbers MK270510, MK270511, MK270512 and MK270513. MHC data are available from the Dryad Digital Repository: 10.5061/dryad.vq05124.

## Supplementary information

Supplementary Material
