# Supplementary material for: MHC class I diversity predicts non-random mating in Chinese alligators (Alligator sinensis)
Source: Heredity (Edinb). 2019 Jan 22;122(6):809–18. doi: 10.1038/s41437-018-0177-8 (PMC6781117; doi:10.1038/s41437-018-0177-8)
Supplement: Supplementary file 1 — Supplementary Material [file 41437_2018_177_MOESM1_ESM.docx]

**Supplementary Material for**

**MHC class I diversity predicts non-random mating in Chinese alligators (*Alligator sinensis*)**

**Qun-Hua Han**^1^, **Ru-Na Sun^1^**, **Hai-Qiong Yang^1^**, **Zhen-Wei Wang^2^**, **Qiu-Hong Wan^1^** **and** **Sheng-Guo Fang^1^ ***

^1^The Key Laboratory of Conservation Biology for Endangered Wildlife of the Ministry of Education and State Conservation Center for Gene Resources of Endangered Wildlife, College of Life Sciences, Zhejiang University, Hangzhou 310058, China

^2^Changxing Chinese Alligator Nature Reserve, Changxing 313100, China

Table S1: Data of adult Chinese alligators collected from three breeding ponds

| Pond name | Candidate father | Candidate mother |
| --- | --- | --- |
| Mixed I breeding pond | 34 | 49 |
| Mixed II breeding pond | 5 | 24 |
| Reconstructed breeding pond | 20 | 50 |
| Total | 59 | 123 |

| Table S2: Summary details of microsatellite loci and MHC class I loci used in this study | | | | |
| --- | --- | --- | --- | --- |
| Loci ID | Repeat motif | Primer sequence | Annealing temperature (°C) | Allele size (bp) |
| CXA-4 | TGGGC(25) | F: AGTTAAATCAGAATCCCCCAGCAT | 58 | 123 128 |
|  |  | R: AATCTCCCAACTCCTCATGGCTA |  |  |
| CXA-6 | TTGGT(20) | F: AAGGCATAACTGATCTTGGCTGAC | 55 | 86 91 |
|  |  | R: AGTGTTGTTCTGGAAAATTCAGGG |  |  |
| CXA-9 | CTTCT(30) | F: CACTTAATGAGTTTTGGGGACCTG | 56 | 102 107 117 |
|  |  | R: GTGGTGAGCTACAGTTCTGGTGAA |  |  |
| CXA-34 | TTTG(24) | F: GTAGATTGCACAAATGCCTTAGCA | 58 | 138 142 146 |
|  |  | R: TGCAGTCTCAAATGCAGATAGGTC |  |  |
| CXA-35 | ACAA(36) | F: AGCTCTGTCTTGAGGAGGCAATAC | 60 | 110 114 118 |
|  |  | R: CAGTCCCTGGTGTAATACTGGCTT |  |  |
| CXA-37 | CTAT(24) | F: ATCCAAAGTCTGACGTGCAAATAA | 56 | 151 155 |
|  |  | R: CCTAGTCAAGGGCAAGGCTATTTT |  |  |
| CXA-39 | ATCT(28) | F: TAAGCAATTTCCCTGACCTATCCA | 58 | 160 164 |
|  |  | R: TTTTTAGTGTGCTTTGAGCTGCC |  |  |
| CXA-41 | ATCT(28) | F: TTTTTATCAGATGGGGCTCAGATG | 58 | 156 160 |
|  |  | R: ATTCATAACTTAGGGGTTGGCAGC |  |  |
| CXA-43 | TATC(36) | F: ATCGAAAAGAAGATTTTCCAAGCC | 55 | 113 117 129 |
|  |  | R: TAGGGCTACAGGCAATCTGAAAAC |  |  |
| CXA-135 | AGC(15) | F: AAATTTTCATTTCTGGGTAAGCCAC | 55 | 140 143 |
|  |  | R: TGACCCTCAATAAACATGCTCAGA |  |  |
| CXA-136 | TAA(30) | F: GTATTCTGCCATTTGGAGGCTACT | 55 | 120 123 126 |
|  |  | R: TTAAGCTGGTGCTCATTATTGCTG |  |  |
| CXA-138 | TGT(18) | F: TTCCTTTTTGCCTCTAGTTTGTGG | 58 | 142 145 |
|  |  | R: ACCTGAAGTCAGTACAAGGGTTGC |  |  |
| CXA-142 | AAC(15) | F: AGTGTGGGATAGGGGGACTCTAAG | 55 | 131 134 |
|  |  | R: CTTTGTGACAGAAATGTCCACCAG |  |  |
| I1327 exon 3 | — | F: AGGGGCCTGGATCTGTGTTTG | 63 | — |
|  |  | R: CCTGGTTAGTGCTGCCGTTA |  |  |
| I20 exon 2 | — | F: GCCCGCTAGTGCTGACCATC | 63 | — |
|  |  | R: CCTGCTCACACCTGCCTGCTA |  |  |

| Table S3 Characteristics of microsatellites and MHC of 182 adult Chinese alligators | | | | |
| --- | --- | --- | --- | --- |
| Locus | No.of Allele | Allele A | Allele B | Allele C |
| cxa-4 | 2 | 123(0.414) | 128(0.586) |  |
| cxa-6 | 2 | 86(0.702) | 91(0.298) |  |
| cxa-9 | 3 | 102(0.163) | 107(0.809) | 117(0.028) |
| cxa-34 | 3 | 138(0.495) | 142(0.196) | 146(0.309) |
| cxa-35 | 3 | 110(0.055) | 114(0.898) | 118(0.047) |
| cxa-37 | 2 | 151(0.457) | 155(0.544) |  |
| cxa-39 | 2 | 160(0.525) | 164(0.475) |  |
| cxa-41 | 2 | 156(0.484) | 160(0.517) |  |
| cxa-43 | 3 | 113(0.514) | 117(0.385) | 129(0.102) |
| cxa-135 | 2 | 140(0.805) | 143(0.195) |  |
| cxa-136 | 3 | 120(0.280) | 123(0.640) | 126(0.080) |
| cxa-138 | 2 | 142(0.613) | 145(0.387) |  |
| cxa-142 | 2 | 131(0.374) | 134(0.626) |  |
| I1327 exon 3 | 2 | A(0.692) | B(0.310) |  |
| I20 exon 2 | 2 | A(0.294) | B(0.706) |  |

Table S4: Relationship between three microsatellite heterozygosity indices

| Index | IR | HL | SH |
| --- | --- | --- | --- |
| IR | - | -0.9578 | 1.0000 |
| HL | -0.9578 | - | -0.9580 |
| SH | 1.0000 | -0.9580 | - |
| Abbreviations: IR, internal relatedness; HL, heterozygosity by locus; SH, standardized heterozygosity. | | | |

Table S5: Relationships between three relatedness indices

| Estimator | Wang | Queller & Goodnight | Lynch & Ritland |
| --- | --- | --- | --- |
| Wang | - | 0.864 | 0.791 |
| Queller & Goodnight | 0.864 | - | 0.944 |
| Lynch & Ritland | 0.791 | 0.944 | - |


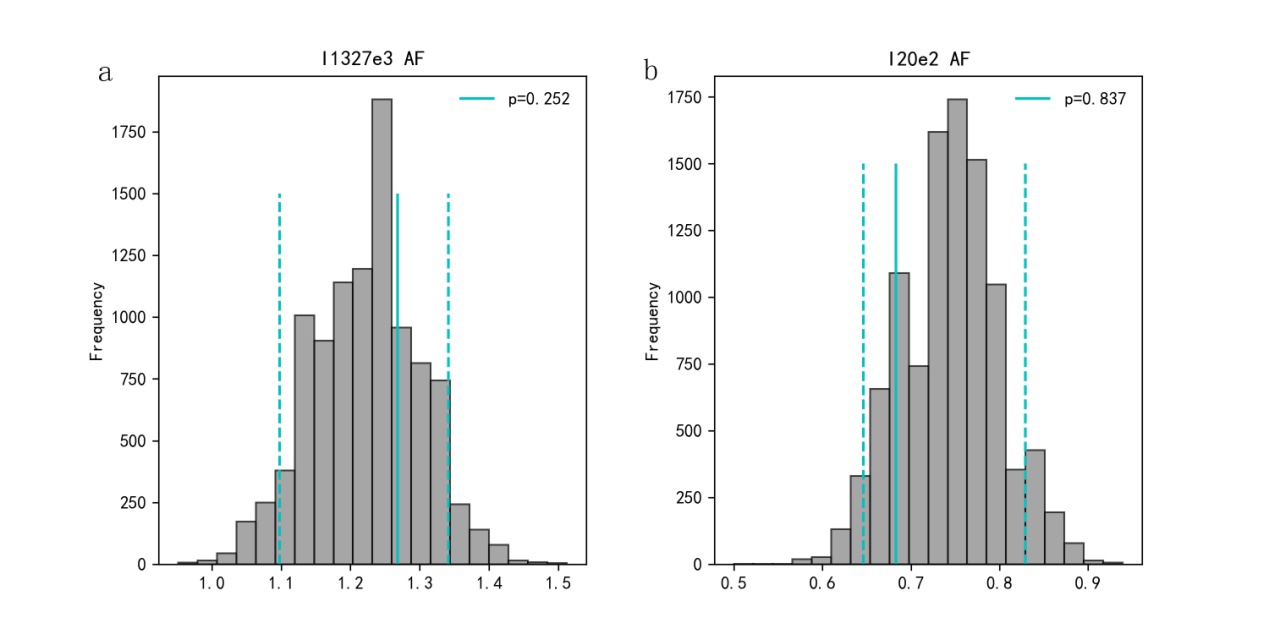


Figure S1: MHC-dependent mate choice tested with Monte Carlo simulations. Frequency distributions of mean allele frequency (AF) of MHC I, generated from 9,999 Monte Carlo simulations of potential males (grey bars) compared with the observed values (solid vertical lines). Two-tailed 95% CI (dashed lines) indicate cut-offs for significant departures from randomly sampled males. (a), AF of I1327e3 loci; (b) AF of I20e2 loci.
